# Physiological and Physical Effects of Different Milk Protein Supplements in Elite Soccer Players

**DOI:** 10.2478/v10078-011-0072-3

**Published:** 2011-12-25

**Authors:** Pablo Christiano Barboza Lollo, Jaime Amaya-Farfan, Luciano Bruno de Carvalho-Silva

**Affiliations:** 1 Department of Food and Nutrition, School of Food Engineering, University of Campinas, Brazil

**Keywords:** whey protein, protein hydrolyzates, casein, physical performance, soccer athletes, BCAA

## Abstract

Brazilian soccer championships involve a large number of teams and are known to cause stress and loss of muscle mass besides other negative physical consequences. This study was designed to compare the effects produced by three types of protein supplements on body composition, biochemical parameters and performance of a top Brazilian professional soccer team during an actual tournament. Twenty-four athletes assessed as having a normal nutrient intake were divided into three groups according to supplementation. Immediately after each daily training, the athletes received 1 g × kg^−1^ of body weight × day^−1^ of either whey protein (WP), hydrolyzed whey protein (HWP) or casein (CAS) for eight weeks. Before and after the experimental period, anthropometric characteristics, physical performance by the yo-yo and 3000m tests, and several biochemical variables in blood (uric acid, total cholesterol, HDL-cholesterol, creatinine, glucose) were measured. While no improvement in physical performance was observed with regard to the applied treatments, casein supplementation resulted in muscle mass increase (p<0.039), while WP and HWP favoured the maintenance of the initial muscle mass. Moreover, the eight-week intervention was found to cause no abnormalities in biochemical and anthropometric variables monitored, but instead, the intervention showed to be positive in comparison to the adverse anthropometric changes, when no supplementation was made. It was concluded that supplementation immediately after training sessions with any of the three sources of protein during the competitive period is beneficial and safe, as well as capable of sustaining or even increasing muscle mass.

## Introduction

Whey proteins (WP), which have high concentrations of branched-chain amino acids, are known for having a good indispensable amino acid balance, high nutritional quality (Marshall, 2004; Sgarbieri, 2004) and digestibility (Yves et al., 1997), and are therefore collectively marketed as a supplemental nurture for athletes.

Heavy physical exercise significantly increases the daily protein requirement (Lemon et al., 1997; Phillips et al., 1997; Tipton and Ferrando, 2008). Branched-chain amino acids (BCAAs) are nutritionally indispensable amino acids that are oxidizable in skeletal muscle, especially as a result of physical exercise. Shimomura et al. (2004) reported that exercise-stimulated burning of BCAAs also increased the requirements of these amino acids in rats. Furthermore, it was shown that BCAA supplementation before and after exercise may promote muscle protein synthesis and contribute to diminish exercise-induced muscle damage (Shimomura et al., 2004).

Energy expenditures of average weight non-professional male soccer players were cited to range between 5.2 and 13 kcal × min^−1^ (Drust et al., 2007). Estimates of distances covered, according to Brazilian standards and by playing position (Barros et al., 2007), are as follows: external defender (10642 ± 663 m), central midfielders (10476 ± 702 m) and external mid-fielders (10598 ± 890 m), followed by forwards (9612 ± 772 m) and central defenders (9029 ± 860 m). The exercise intensities vary greatly, including walking, or jogging (5537 ± 263 m), followed by moderate-speed running (1731 ± 399 m); low speed running (1615 ± 351 m); high-speed running (691 ± 190 m) and sprinting (437 ± 171 m).

Tipton et al. (2004) compared the acute anabolic response of milk whey proteins (fast proteins) in humans with that of the caseins (slow proteins) during resistance exercise in short trials finding no difference between these two types of proteins. Recent findings, however, suggest that whey protein hydrolyzates appear to have biochemical properties far beyond those expected from the facilitated digestion and absorption such as cell signalers, thus modifying metabolism and causing significant physiological changes (Morifuji et al., 2009)

Protein or amino acid supply is closely associated with muscle mass gain or loss (Biolo et al., 1995; Roth, 2008), and oral solutions are a practical way of delivering supplements designed to promote muscle protein anabolism in exercising humans (Tipton et al., 1999). A number of advantages, such as increased muscle glycogen stores, immune modulation, among others, have been attributed to the milk whey proteins, either as part of the diet or as supplements in both animals and humans (Ha, E and Zemel, 2003; Pimenta et al., 2005; McIntosh et al., 1998). The milk whey proteins, or whey proteins (WP), have high concentrations of the readily metabolized branched-chain amino acids, and are known for having a good indispensable amino acid balance, high nutritional quality (Marshall, 2004; Sgarbieri, 2004) and digestibility (Yves et al., 1997).

Beyond the benefits that whey proteins offer, once the process of hydrolysis commences, key peptides are generated, and such products become involved in extra functions (Meisel, 1998). Consequently, whey protein hydrolyzates have been reported to offer additional advantages, including antioxidant properties and magnified glycogen stores above the levels obtained from the unhydrolyzed whey, most likely because of the bioactive peptides they contain (Morifuji, 2009; Nery-Diez et al., 2010; Peng et al., 2009).

Protein or amino acid supply is closely associated with muscle mass gain or loss (Roth, 2008) and oral solutions are a practical way of delivering supplements designed to promote muscle protein anabolism in exercising subjects (Tipton et al., 1999). Although both physiological and nutritional benefits have been shown to occur under laboratory conditions and with non-athletes (Bolster et al., 2005; Borsheim et al., 2002; Esmarck et al., 2001; Miller et al., 2003; Tipton et al., 1999; 2003; 2007), very little is known of how elite athletes respond to medium or long term supplementation during strenuous training and competition.

In this study, we have compared the response of professional Brazilian soccer players of the top national division produced by three different protein supplements, common whey protein, hydrolyzed whey protein and casein in an 8-week intervention. The monitored variables included standard anthropometric parameters, body composition, physical performance along the season and plasma concentrations of uric acid, glucose, creatinine, total and HDL-cholesterol.

## Material and Methods

Twenty-four male young, professional soccer players, engaged in the competitive task of the first Brazilian league, mean age of 19 ± 1.4 years, successfully qualified to participate in the study. All subjects were members of a professional team of São Paulo State and were engaged in an official tournament, along with 47 other teams. The subjects were informed about all procedures and possible benefits and risks involved in this research before a written consent to participate was handed out. The study was approved by the University of Campinas’ Ethics Committee.

The work load was on average one or two games, and between six and eight training sessions per week. Each training (technical-tactic) session lasting approximately 2.5 hours, was carried out directly on the field. In order to fulfil the six to eight sessions per week, two sessions normally took place during the same day.

Taking into consideration their corresponding positions in the field, the athletes were randomly divided into three groups and their standard institutional diets supplemented with either whey protein concentrate (WP; 91.4% protein, n = 8), hydrolyzed whey protein (HWP 87.0% protein; degree of hydrolysis = 10.5%, n = 8) or casein (CAS; 88.6% protein, n = 8), all from New Zealand Milk Products, Wellington N.Z.

Each subject in every group received a bottle containing 1g of protein per kg of body mass/day immediately after the daily training session in a double-blind manner during the experimental period. Relative intensity and training volume were standardised for all athletes having the same role in the field. Given that the professional institution did not allow the authors to carry a group without supplementation, the data with which a comparison could be established were collected during the period immediately preceding the championship session (Table 1). The adaptation period, comprising the first eight weeks of the tournament, is a phase that includes daily training during which the athletes substantially improve their physical conditioning and body composition (Häkkinen et al., 2000).

The adequacy of nutrient and energy intakes of the athletes was assessed using the DRI (Dietary Recommended Intakes) tables and the nutrient planning program Dietpro 4.0 (Belo Horizonte, Brazil). After each athlete’s food and nutrient ingestion was estimated, the mean supplemental dosage of every group was calculated in order to provide 2.3 g/kg^−1^ × day^−1^ body mass, based on an estimate of 15% of the total energy value (WHO/FAO, 2003). All groups were housed and fed under the same conditions, supervised by a dietician, and tendered by the same dining services of the sports institution. The level of protein supplementation was decided upon the recommendation for high performance athletes (Lemon et al., 1997). Athletes were strongly advised not to have any meals outside the institution, but if they did so, reports were made to the nutritionist in order to make adjustments. The athletes were also prohibited to consume any other nutritional supplement during the test period.

Mean anthropometric data as assessed between 8 a.m. and 10 a.m. with the athletes in the fasted state are shown in Table 2. All measurements were made before and after the experimental cycle in the resting state according to established procedures. Prior to the beginning of the supplementation intervention, anthropometric and nutritional data of the athletes hosted by the soccer institution were collected by physical educators and nutritionists for five consecutive years. Although these data consistently showed significant muscle mass losses at the end of every tournament, only data of the fifth year (Table 1) were used as reference to assess the impact of supplementation.

Skinfold thickness was measured to the nearest 0.5 mm with a calibrated Lange calliper at nine anatomical sites by the same trained technician. The nine sites measured were chest, sub scapula, mid-axillary, supra-iliac, iliac crest, abdomen, triceps, thigh and calf. All measurements were taken on the subject’s right side. The body composition was determined using the set of equations proposed by Slaugter et al. (1988) and the arm-transverse, arm-muscle and arm-fat areas (ATA, AMA and AFA) were determined according to Frisancho (1981).

Performance variables were monitored before, in the middle of the season, and at the end of the supplementation, which was the end of the season. The physical tests were a recovery yo-yo test, level 2, and a 3000-m time trial. The application of both tests followed the recommendations of Bangsbo et al. (2008) and the American College of Sports Medicine (2006).

The yo-yo tests were performed as described by Brandley et al. (2011), and consisted of repeated 20-m shuttle runs at progressively increasing speeds dictated by an audio beep emitted from a CD player. Between each shuttle, players had a 5-s period of jogging around a mark placed 2.5 m behind the finishing line. Failure to perform the shuttle run in time on two consecutive attempts resulted in termination of the test and the distance covered in the last complete successful shuttle was recorded as the test result. All testing sessions were performed in the stadium. The 3000-m endurance tests were performed on a 400-m track.

The physical, biochemical and anthropometric assessments were limited to the least invasive tests, the only ones allowed by the sports institution because of potential impingement on the athletes’ performance during the competitive season. Forty-eight hours after the last training session, of the last competitive microcycle, blood samples (10mL) were collected in the fasted state by venipuncture (heparin-containing Vacutainers), kept at 0 °C, and centrifuged at 3000×g (4 °C, 10min) to obtain plasma.

For the assessment of sera, uric acid, total cholesterol, HDL-cholesterol, creatinine and blood glucose, standard enzymatic spectrophotometric determinations were performed employing the Laborlab (Campinas, Brazil) kit on an automated analyzer (DuPont ACA-IV and Hitachi 747-100).

The SPSS 11.5.0 software was used to conduct all statistical analyses and results were expressed as means and SEM. Descriptive statistics for the dependent variables were computed using the SPSS means procedure. The ANOVA and post hoc Tukey tests were used, the level of significance was set at p ≤ 0.05.

## Results

Age, body mass and body height (Mean ± SEM) were 19 ± 1.4 years, 74.4 ± 6.44 kg and 181.5 ± 0.62 cm, respectively. All players had more than three years of systematic training in soccer and were accommodated in the team’s quarters during the entire experimental period, consuming a controlled diet, following the same sleeping and training schedules. During road games the identical control was maintained. The experiment was performed while the athletes were participating in the country’s main tournament. The team ranked second at the end of the championship.

The average energy intake of the athletes was 15,974 kJ, distributed into 55% carbohydrates, 17.8% protein and 27.2% lipids, similar to those estimated elsewhere for soccer athletes (16,400 on training, and 15,580 kJ on match days; (Rico-Sanz, 1998)). The individual mean total protein intake (dietary plus supplementation) was 2.3 ± 0.3 g × kg^−1^ × day^−1^, varying between 2.0 and 2.5.

### Anthropometric characteristics

The pre- and post-supplementation variables are shown in Table 2. After eight weeks of supplementation, an increase of muscle mass was detected only in the casein group (p < 0.039). Meanwhile, the group supplemented with WP had a small (5%), but significant (p < 0.048) decrease in AMA, and a 27% increase of AFA, while an increase of BFM of 4.7% in the HWP group was observed.

### Physical Tests

The effects on performance resulting from the different types of supplementation are shown in Table 3. No significant variation (lowest *p* = 0.180) in either physical performance or lactate concentrations was observed due to the supplementation.

### Biochemical Tests

Among the four monitored variables, creatinine, uric acid, glucose and HDL-cholesterol, significant increases were observed only for creatinine (*p* = 0.021) and uric acid (*p* = 0.028) in the group supplemented with WP (Table 4).

## Discussion

No side effects were observed due to the intervention with the protein supplementation in any of the groups in spite of the fact that the total protein intake (2.3 g × kg^−1^ × day^−1^) exceeded recommendations for endurance athletes (Tipton et al., 2004). Uric acid was found to be about 15% increased and creatinine about 8% higher in the group that consumed the WP supplement. Although the alterations did not represent a departure from normal ranges (Clifford et al., 2011), this result should not be considered necessarily as negative. Uric acid is known to have some beneficial properties. Waring et al. (2003) found that high uric acid concentrations are associated with increased serum antioxidant capacity and reduced oxidative stress during acute physical exercise in healthy subjects, confirming the biological importance of uric acid in vivo.

Creatinine is a nitrogen metabolite, which is widely recognized as a marker of the metabolic rate, protein intake and renal function. The creatinine level in the WP group was slightly raised, but still remained in the middle of the range of normality (0.70 – 1.20 mg × dL^−1^; Ceriotti et al., 2008). Altogether, the modest variations of these two parameters indicated that the renal function was not altered.

Total and HDL-cholesterol, of which values might have increased with regard to aerobic physical activity (Leaf, 2003), high protein intakes (Dusmenil et al., 2001), as well as the type of protein ingested (Minehira et al., 1999), were unaltered, together with the glycaemic levels, which also remained constant and normal.

In addition to the known role of exercise and protein supplementation on the possible gain of skeletal muscle mass (Blake et al., 2000; Roth, 2008), our data suggest that supplementing immediately after training with milk proteins can provide significant benefit for maintaining, or even gaining muscle mass in young elite athletes undergoing intense and prolonged physical exercise. It should be remembered that the net gain was only associated with the casein treatment. These results acquire an important perspective when compared with the muscle mass losses that were historically observed in elite soccer athletes of this sports institution, particularly during the most recent tournaments prior to the intervention, when the young athletes were not receiving any protein supplements and yet the same nutritional care and medical control practices were observed throughout the season.

Furthermore, the present data show that, as it has been reported for non-athlete volunteers (Blake et al., 2000, Bolster et al., 2005; Borsheim et al., 2002; Esmarck et al., 2001, Phillips et al., 1997), systematic protein supplementation can also modulate skeletal muscle protein net gain in high performance young athletes engaged in an actual championship, if done daily immediately after every endurance task, and in which the protein source is an important factor for net muscle mass gain.

Although our data did show an improvement in body composition of the athletes that consumed the casein supplement, this did not result in an improvement in either of the yoyo or the 3000-m tests. The anaerobic and aerobic tests were included to verify in athletes if the hydrolyzate conferred an advantage in performance as observed earlier in our laboratory with rats consuming the same three sources of protein, but at lower protein intakes (Pimenta et al., 2006; Nery-Diez et al., 2010). The outcome, however, could be explained considering that two performance tests were carried out on individuals consuming the protein supplements already at high loads, with a concomitant loss of efficiency.

Also, the slight total body fat increase in the group supplemented with HWP did not represent an unfavourable result due to the fact that this group had a mean initial BFM below the average of the other two groups, and the volunteer selection followed the positions in the field.

Regarding the acute phase of supplementation, an anabolic effect has been reported (Tang et al., 2009) to occur in young volunteers, consuming a single 10 g supplement of either casein, whey protein isolate or soy protein isolate, immediately after a session of resistance exercise. The authors found that the hydrolyzate had superior (greater than two-fold) anabolic response than that of casein. In our study, however, the young volunteers consumed 75g of casein (1 g × kg^−1^ of body mass), for eight weeks, showing a clear increase in muscle mass from casein, while the other proteins were still effective in maintaining muscle mass. At first, the difference between these two results could be explained by the widely differing dosages and possibly different efficiencies of utilization. Protein supplementation studies with humans under laboratory conditions exist (Bolster et al., 2005; Tipton et al., 1999; 2001; 2004; 2007), but not with elite athletes during a competitive season, when the demands of matches repeatedly take the subjects to their physical and emotional limits.

Protein supplementation is known to cause an anabolic response when in combination with exercise. Tipton et al. (2004) reported about the insulinotropic response of humans consuming either whey or casein protein supplements, showing that the whey protein can stimulate the secretion of insulin three times higher than the extent stimulated by casein. Meanwhile, Power et al. (2009) found that the whey protein hydrolyzate had a stimulating power greater than that of the unhydrolyzed protein.

As suggested by other scientists in various types of sport disciplines, the data collected from this group of Brazilian elite soccer players show that during the competitive season protein supplements would be in order if attainment of the players’ superior physiological demands is expected, and the type of protein used as a supplement may have a significant influence on the athlete’s ability to maintain, or perhaps increase muscle mass.

Supplements of 1g per kg of body mass consumed immediately after the training sessions by young male elite athletes appeared to be a safe strategy to follow during soccer tournament periods of several weeks aiming at muscle mass conservation. In view of these results and supporting literature, coaches could consider protein supplementation, particularly with the whey protein hydrolyzate as a potentially beneficial nutriment for the young athlete facing intense and prolonged physical training in order to maintain an adequate body composition all throughout. In this type of sport, training is not aimed at muscle mass building beyond an ideal point, which should be attained prior to the competitive period. According to our results (1 g × kg^−1^ body mass), however, casein could be better than the whey proteins, whether whole or hydrolyzed, when the athlete has a below-ideal muscle mass.

## Figures and Tables

**Table 1:** *Muscle mass evolution of the athletes in the preceding championship and in preceding four years. The initial measurement refers to the middle of the championship period*.

Variable	Preceding four years n=54		Preceding championship n=17	
	
	Initial	End	Initial	End
	
	Mean (±SE)		Mean (±SE)	
	
BFM %	12.15(0.2)	12.48(0.2)	12.55(0.7)	12.69(1.1)
MM	34.05(0.3)	32.6(0.3)^*^	34.72(2.1)	33.13(2.3)^*^
BM (kg)	71.6(0.7)	70.3(0.7)^*^	73.01(2.7)	71.71(2.5)^*^
BMI	22.6(0.2)	22.1(0.2)	22.3(0.9)	21.86(0.8)
ATA	77.12(0.4)	75.2(0.4)^*^	79.45(3.4)	76.42(3.8)^*^
AMA	67.6(0.3)	66.7(0.3)	68.2(2.1)	66.4(2.2)
AFA	9,5(0.2)	8,5(0.2)^*^	11.25(0.6)	10.02(0.7)
Σ7 skinfolds ^1^	60,41(0.3)	62,7(0.3)^*^	62.12(2.9)	63.27(3.7)^*^

BFM %, body fat mass percent; MM, muscle mass; BM, body mass; BMI, body mass index; ATA, arm transverse area, AMA, arm muscle area; AFA, arm fat area;

* p<0.05 between beginning and end of championship.

^1^ - mm.

**Table 2:** *Mean of anthropometric characteristics of subjects in the three groups before and after supplementation*.

Variable	Before supplementation Mean (±SE)			After supplementation Mean (±SE)		

	CAS	WP	HWP	CAS	WP	HWP
	
BFM %	13.54(1.3)	12.73(1.1)	9.7(0.4)	13.04(0.7)	12.33(0.9)	10.16^*^(0.5)
MM (kg)	37.11(2.7)	33.77(1.9)	36.33(2.2)	38.16^*^(1.7)	34.35(2.9)	36.79(1.9)
BM (kg)	80.25(4.9)	72.02(2.7)	72.84(3.6)	77.97(3.8)	71.08(−4)	73.67(3.9)
BMI (kg/m^2^)	23.96(1.4)	22.78(1.9)	22.65(1.7)	23.28(1.7)	22.48(−1)	22.91(1.3)
ATA (cm^2^)	80.07(6.1)	80(−6)	81.28(2.4)	81.28(7.1)	79.17(6.3)	81.24(5.5)
AMA (cm^2^)	69.14(3.7)	69.27(5.2)	70.98(3.2)	70.3(3.5)	65.52^*^(3.2)	70.39(3.5)
AFA (cm^2^)	10.93(−1)	10.73(0.7)	10.3(0.7)	10.98(0.5)	13.65^*^(1.3)	10.85(0.8)
Σ7 skinfolds^1^	65.1(3.8)	56.4(5.5)	54.1(3.1)	62.2(5.9)	54.2(4.3)	63.6^*^(3.8)

BFM %, percent body fat mass; MM, muscle mass; BM, body mass; BMI, body mass index; ATA, arm transverse area, AMA, arm muscle area; AFA, arm fat area;

* p < 0.05 when comparing before vs. after supplementation.

1 mm.

‡ The players were grouped taking into consideration each one’s position in the field in order to avoid selecting more than one player of the same position in any one group.

**Table 3:** Physical performance of the elite soccer players before and after supplementation.

Tests		Before supplementation			After supplementation		
	
		CAS(±SE)	WP (±SE)	HWP(±SE)	CAS (±SE)	WP (±SE)	HWP (±SE)
	
Yo-yo test	Distance^1^	434(38)	446(32)	383(31)	510(36)	440(19)	413(19)
	Lactate^2^	10.3(0.7)	10.8(0.5)	9.8(0.5)	10.7(0.8)	10.2(0.6)	10.3(0.06)
3000m	Time (sec)	728(41)	739(63)	743(26)	723(44)	722(33)	745(33)
	Lactate^2^	8.59(0.5)	7.86(0.6)	8.27(0.6)	9.2(0.5)	8.26(0.6)	8.52(0.6)

^1^ – in meters.

^2^ – in (mmol/L)

**Table 4:** Biochemical variables for the three groups of athletes before and after supplementation (mean ± SEM).

Variables	Before supplementation			After supplementation		
	
	CAS (±SE)	WP(±SE)	HWP(±SE)	CAS (±SE)	WP(±SE)	HWP(±SE)
		
Creatinine†	0.75 (0.03)	0.71(0.03)	0.85(0.03)	0.79(0.03)	0.82 (0.05)*	0.83 (0.07)
Uric Acid †	5.26 (0.16)	4.78(0.22)	5.38(0.21)	5.49(0.24)	5.2 (0.39)*	5.62 (0.18)
Cholesterol†	162.2 (1.5)	172(1.4)	160.2(1.5)	168.1(1.5)	166.5(1.4)	154.1(1.4)
HDL†	46.57 (1.2)	42.8(1.2)	39.9(1.1)	48.2(1.3)	45.3(1.2)	43.4(1.3)
Glucose†	78.6 (1.7)	82.26(1.6)	83.4(1.7)	83.2(1.7)	81.5(1.7)	84.1(1.6)

† Units expressed in mg/dL;

* p<0.05 when comparing before vs. after supplementation.
